# Dietary assessment of ochratoxin A in Chinese dark tea and inhibitory effects of tea polyphenols on ochratoxigenic *Aspergillus niger*

**DOI:** 10.3389/fmicb.2022.1073950

**Published:** 2022-12-06

**Authors:** Yi-qiao Zhao, Wen-bao Jia, Si-yu Liao, Lin Xiang, Wei Chen, Yao Zou, Ming-Zhi Zhu, Wei Xu

**Affiliations:** ^1^College of Horticulture, Tea Refining and Innovation Key Laboratory of Sichuan Province, Sichuan Agricultural University, Chengdu, China; ^2^Key Laboratory of Tea Science of Ministry of Education, National Research Center of Engineering Technology for Utilization of Functional Ingredients From Botanicals, College of Horticulture, Hunan Agricultural University, Changsha, China

**Keywords:** hazard quotient, Ochratoxin A contamination, risk assessment, polyphenols, non-ribosomal peptide synthetase

## Abstract

In recent years, there has been an increasingly heated debate on whether Chinese dark tea is contaminated with mycotoxins and whether it poses health risks to consumers. In this study, a rapid method based on high-performance liquid chromatography was used to detect ochratoxin A (OTA) in Chinese dark tea samples from different regions of China and different years. Of the 228 Chinese dark tea samples tested, 21 were detected for OTA contamination, with a concentration ranging from 2.51 ± 0.16 to 12.62 ± 0.72 μg/kg. Subsequently, a dark tea drinking risk assessment was conducted, and the hazard quotient for each group was far below the acceptable level of 1.0. Of the 12 *Aspergillus* spp. strains isolated, one strain of *Aspergillus niger* had the ability to produce OTA. We also found that tea polyphenols and epigallocatechin gallate inhibited the growth of ochratoxin-producing *Aspergillus niger* and the expression of non-ribosomal peptide synthetase *(NRPS)*, a key gene for ochratoxin synthesis. Thus, OTA contamination of dark tea is at an acceptable risk level, and the inhibition of ochratoxigenic *Aspergillus niger* by polyphenols provides new insights into the safety of dark tea consumption.

## Introduction

Dark tea, which is highly preferred in China, is a unique type of solid fermentation tea (Wang et al., 1991). There are various types of dark tea available in different regions of China, and the major types are Kang brick tea in Sichuan, Fu brick tea in Hunan and Shaanxi, Ripe Pu-erh tea in Yunnan, and Qing brick tea in Hubei (Wang R. et al., 2018). The unique flavor qualities and health benefits of dark tea are attributed to pile-fermentation, which is a special process in dark tea processing. *Aspergillus niger* is the dominant fungus in the pile-fermentation processing (Ou, 2017; Xiong, 2017), and parts of *Aspergillus niger* have the ability to produce ochratoxin A (OTA; Sánchez-Hervás et al., 2008; Iqbal et al., 2014; Wang et al., 2016; Deng et al., 2020). Recent reports have shown that *Aspergillus niger* isolated from dark tea can produce OTA. This implies that there is a possibility of dark tea contamination by OTA (Zhou et al., 2015; Zhao et al., 2021).

With the increasing consumption of dark tea, the study of OTA determination and risk assessment for dark tea is imminent. Numerous studies (Haas et al., 2013; Liu et al., 2016, 2017; Mo et al., 2016; Ye et al., 2020) regarding OTA determination in dark tea have been conducted, the concentrations ranging from 0.22 to 94.7 μg/kg, but a larger number and more representative samples of dark tea should be investigated. Risk assessment of OTA in dark tea has been carried out in some studies (Ye et al., 2020; Yan et al., 2021), and the results showed that there is no observed risk concern to consumers as the hazard quotient (HQ) was below 1.0. In addition, large amounts of tea consumption data are urgently required to generate a more accurate assessment.

Ochratoxin A, a group of secondary metabolites produced by the fungi *Aspergillus* (Wang Y. et al., 2018), is nephrotoxic, hepatotoxic, immunotoxic, genotoxic, neurotoxic, and teratogenic (Chen et al., 2018). This compound is widely found in cereal products, wine, coffee, tea, and even meat products (Duarte et al., 2010; Kumar et al., 2020). OTA contamination is an increasing concern worldwide because of its harmful effects on human health. The cluster of genes involved in OTA synthesis in *Aspergillus niger* has been extensively investigated, and the results suggest that polyketide synthase (*PKS*), non-ribosomal peptide synthetase (*NRPS*), cytochrome p450 (*P450*), basic leucine zipper (*BZIP*), and halogenase (*HAL*) may be key genes of the OTA synthesis gene cluster in *Aspergillus niger* (Karolewiez and Geisen, 2005; O’Callaghan et al., 2003; Pel et al., 2007).

Previous studies (Zhang et al., 2016; Zhao et al., 2021) have shown that OTA-producing fungi in dark tea are characterized by various fungal species and high detection frequencies; however, the detection rate and content of OTA in dark tea samples were lower than expected. This may be due to the ability of tea polyphenols (TPs) to inhibit the production of OTA by OTA-producing fungi. Many studies have shown that TPs can inhibit fungal production of mycotoxins (Mo et al., 2013; Wu Q., 2013; Li et al., 2015).

In this study, we used a rapid high-performance liquid chromatography (HPLC)-based assay to detect OTA in dark tea samples from different years obtained from six different regions of China. The OTA contamination levels of dark tea obtained in this study and the consumer data on dark tea consumption across the country were used to assess the risk of dark tea consumption. In addition, 12 *Aspergillus* spp. strains were isolated from tea samples containing OTA, and one strain of *Aspergillus niger* had the ability to produce OTA. We also investigated the inhibitory effects of different concentrations of TPs and epigallocatechin gallate (EGCG) on the growth of OTA-producing *Aspergillus niger* (AN3) isolated from tea samples containing OTA, and the mechanism of inhibition of their toxin production by TPs and EGCG.

## Materials and methods

### Materials and reagents

**Figure 1:**
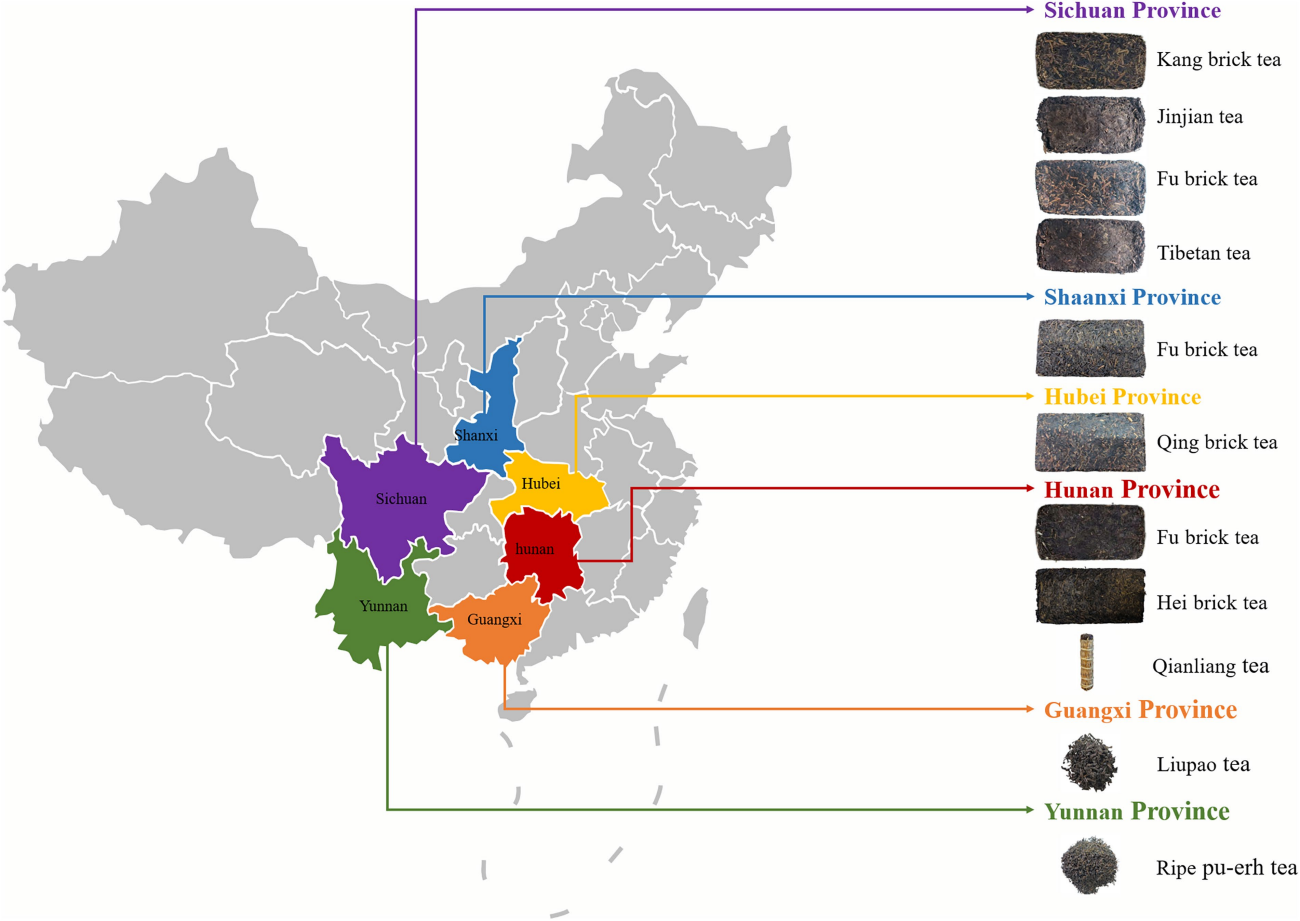
The samples of dark tea.

Kang brick tea from Sichuan, Fu brick tea from Hunan and Shaanxi, Ripe Pu-erh tea from Yunnan, Qing brick tea from Hubei, and Liu Bao tea from Guangxi (Figure 1), with 228 samples of different vintage dark tea that were randomly purchased from tea markets, tea retail outlets, and online shops (March 2021–September 2021). The samples were stored in a clean and air-ventilated environment prior to the analysis.

Ochratoxin α (OTα), OTA, and ochratoxin B (OTB) were purchased from Pribolab Pte Ltd. (Qingdao, China). TPs and EGCG were purchased from Hunan Sunfull Bio-tech Co., Ltd. (Changsha, China). Chromatographic-grade methanol, acetonitrile, and glacial acetic acid were provided by Chengdu Kelong Co. Ltd. (Chengdu, China).

### Sample preparation and HPLC system and operating conditions

The tea sample was prepared by extracting 1.0 g of crushed dark tea with 10.0 ml of a methanol-formic acid (25:1 v/v) solution. The solution was mixed using a stirrer for 2 min and further extracted using ultrasonic bathing for 10 min. It was then centrifuged at 4,000 rpm for 10 min, the supernatant was filtered through filter paper, and the volume was fixed to 4.0 ml. The filtrate was filtered through a 0.45 μm organic filter membrane before HPLC analysis.

The filtrate was measured on an Agilent 1260Infinity II (Agilent Technologies Co. Ltd., Palo Alto, CA, United States) HPLC system equipped with a fluorescence detector.

For HPLC analysis of dark tea, OTA was separated using a C_18_ column (5 μm, 250 × 4.6 mm; Phenomenex, Torrance, CA, United States). Separation was performed at 30°C, and the injection volume was 10.0 μl. The mobile phase was composed of water-glacial acetic acid(98:2 v/v; phase A) and acetonitrile (phase B), and gradient elution was as follows: 0–10 min, 88–80% A, 12–20% B; 10–12 min, 80–70% A, 20–30% B; 12–19 min, 70–50% A, 30–50% B; 19–31 min, 50–0% A, 50–100% B; 31–40 min, 0–88% A, 100–12% B; 40–45 min, 88% A, 12% B.

For the HPLC analysis of the liquid culture, OTα, OTA, and OTB were separated using a Pribolab ODS C_18_ column (2.5 μm, 150 × 4.6 mm), water-glacial acetic acid (98:2 v/v; phase A) and acetonitrile (phase B), and isocratic elution (50:50).

### Method validation

Recovery rates were evaluated by adding three concentrations (5, 10, and 20 μg·kg^−1^) of OTA to non-toxic tea samples. Recovery was determined by comparing the average peak areas of the spiked samples with those of the standard solutions. Linearity was calculated by peak areas and concentrations of the OTA standard (1, 5, 10, 20, and 30 μg·L^−1^) using HPLC. The limit of detection (LOD) and limit of quantification (LOQ) were determined by analyzing the lowest OTA in the tea samples. The LOD and LOQ were calculated based on a signal-to-noise ratio (S/N) of three (3:1) and 10 times (10:1) the background chromatographic noise, respectively.

### Tea consumption questionnaire

Tea consumption data were obtained through a questionnaire with a total of 446 participants, who were selected randomly from Sichuan, Chongqing, Hunan, Guangdong, Yunnan, and Inner Mongolia. The participants were questioned about their sex, age, weight, city, brewing volume, and proportion of drinking volume to brewing volume.

### Health risk assessment

#### Deterministic risk assessment

The dietary risk of mycotoxin exposure from drinking dark tea was assessed based on contamination levels and consumption data. The health neoplastic risk of OTA was assessed based on the HQ (IARC, 1993). A HQ value of <1.0 indicates that the population is not at risk of exposure (WHO, 2005; EFSA, 2013). The average daily intake of ADD (μg.kg^−1^.day^−1^) and the HQ values were calculated using the following equations:


ADD=C*CA/BW

HQ=ADD/RfD

Here, C is the average mycotoxin concentration in dark tea (μg.kg^−1^), CA is the amount of dark tea consumed (g.person^−1^.day^−1^), BW is the average body weight of the population (kg), ADD is the average daily intake of OTA, and RfD is the provisional maximum tolerable daily intake (PMTDI) suggested by the World Health Organization (WHO).

For the risk assessment, average and heavy tea consumers (95% of tea consumers) were evaluated. We used 0 (lower limit), 1/2 LOD (middle limit), and LOD (upper limit; GEMS/Food-EURO, 1995) as the three possible scenarios for the risk assessment. In addition, we explored extreme exposure scenarios, that is, the mean and maximum OTA contamination values of all tea samples contained in this study for neoplastic risk assessment.

#### Probabilistic risk estimation

Deterministic risk assessment methods ignore the variability and uncertainty of data and fail to reflect the differences among individuals. Probabilistic risk assessment compensates for the shortcomings of deterministic risk assessment by quantifying variability and uncertainty and is now the main tool for dietary exposure assessment. Probabilistic assessment requires assessment of all risk factors in the population, such as data on the body weight of all individuals in the assessment population, assessment of tea consumption data, and data on the OTA content of the tea. Therefore, a large data resource is necessary to carry out accurate probabilistic assessments. The variability of exposure factors (i.e., OTA contamination, body weight, and tea consumption) was assessed for each assessment group through Monte Carlo simulations using the Crystal Ball (*Version 11.2*) software to determine the best-fit distribution. Sensitivity analyses were performed using the Crystal Ball software to assess the contribution of each exposure factor to neoplastic risk. A total of 10,000 iterations were performed using the Monte Carlo simulation. The lower, middle, and upper limits as well as the average and maximum (extreme case) contamination exposures were determined separately.

### Isolation of fungal strains in tea samples containing OTA

Fungal strains in tea samples containing OTA from six regions (Sichuan, Hunan, Yunnan, Shaanxi, Guangxi, and Hubei) were isolated. The toxin-producing abilities of the isolated fungal strains were analyzed, and those with toxin-producing abilities were identified by phylogenetic analysis of partial β-tubulin (*BenA*; *Bt2a*:GGTAACCAAATCGGTGCTGCTTTC, *Bt2b*:ACCCTCAGTGTAGTGACCCTTGGC), partial calmodulin (CaM; *CF1L*:GCCGACTCTTTGACYGARGAR, *CF1M*:AGGCCGAYTCTYTGACYGA, *CF4*:TTTYTGCATCATRAGYTGGAC), and RNA polymerase beta gene (*RPB2*; *fRPB2-5F*:GAYGAYMGWGATCAYTTYGG, *fRPB2-7cR*:CCCATRGCTTGYTTRCCCAT), which were then used in subsequent experiments.

### Inhibition of TPs and EGCG on *Aspergillus niger* AN3

#### Growth inhibition

Tea polyphenols and EGCG were added to CYB medium separately so that the concentrations of TPs and EGCG in the medium were 0, 10, 25, 50, and 75 mg/ml. After incubation at 28°C for 7 days, the growth of *A. niger* AN3 was observed.

#### Inhibition of the mechanism of toxicity production

The cultures were expanded at 50 mg/ml concentrations of TPs and EGCG for 3 and 7 days, respectively, and then detected for OTα, OTA, and OTB in the culture broth. RNA of *A. niger* AN3 was extracted, reverse-transcribed, and subjected to RT-PCR.

The relative expression levels of *PKS*, *NRPS*, *P450*, *BZIP*, and *HAL*, the key genes for OTA synthesis in *A. niger* AN3 under TPs and EGCG treatment conditions, were measured, and the β-tubulin gene was used as an internal reference for normalization of gene expression.

### DNA extraction and RNA preparation

DNA of *A. niger* AN3 was extracted using the Fungi Genomic DNA Extraction Kit (D2300, Solarbio, Co., Ltd., China) according to the manufacturer’s instructions. The total DNA was eluted in 100 μl of elution buffer. To measure the expression of *PKS*, *NRPS*, *P450*, *BZIP*, and *HAL*, total RNA was extracted by Total RNA Extraction Kit (Solarbio, Co., Ltd., China). RNA was reverse-transcribed using FastKing RT-qPCR Kit (Tiangen Biotech Co., Ltd., Beijing, China). All the primers for PCR and qPCR were designed by Sangon Biotech Co., Ltd. (Shanghai, China).

### Statistical analysis

All experiments were performed with three replicates. The variability of exposure factors, sensitivity, and the Monte Carlo simulation were performed using the Crystal Ball Version 11.2 (Oracle Corp., Redwood, CA, United States). The significant differences based on one-way analysis followed by the Duncan’s Multiple Range Test were performed using SPSS Statistics Version 25.0 (IBM Corp., Armonk, NY, United States).

## Results

### Method validation

**Figure 2:**
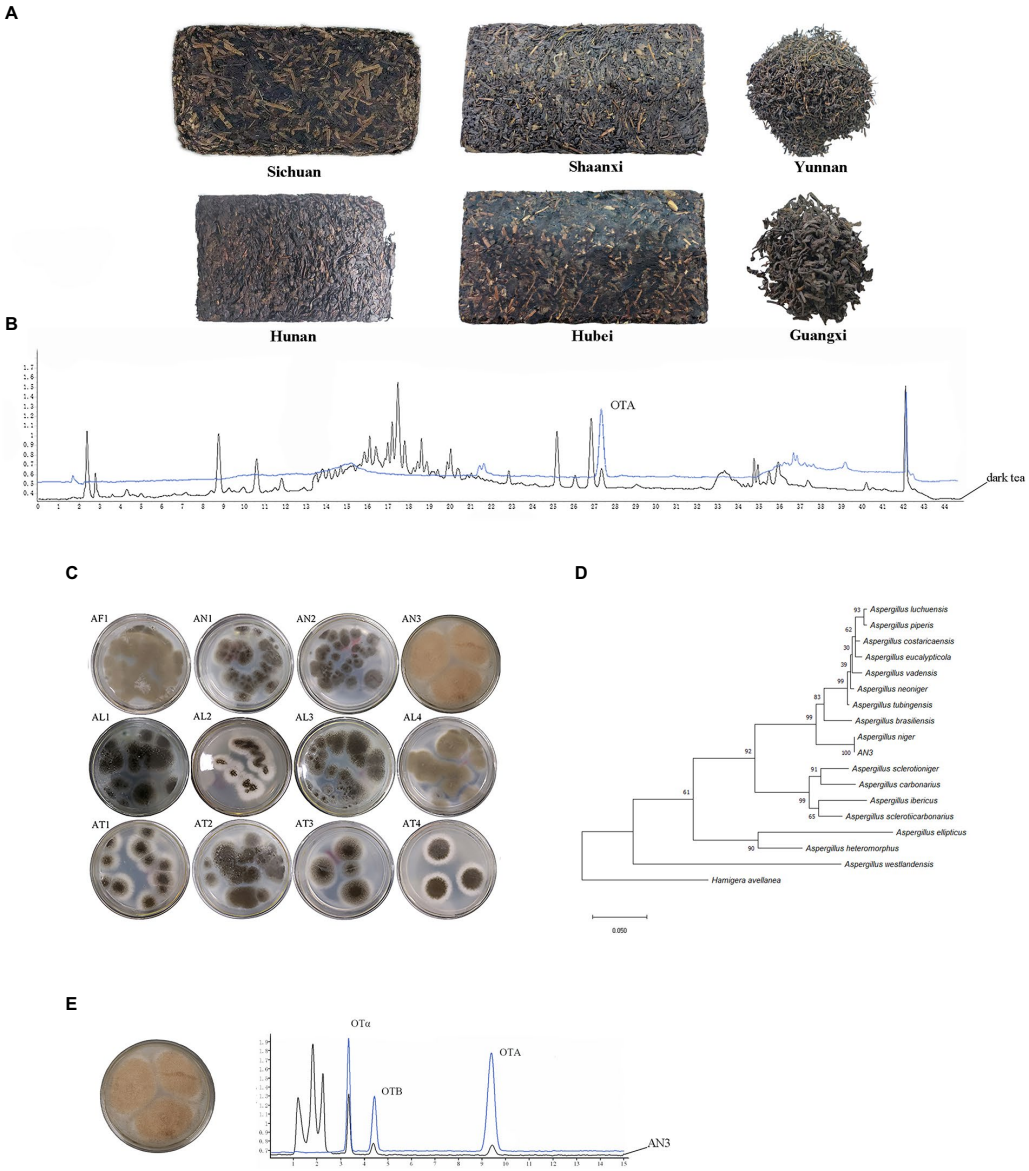
OTA detection in dark tea samples and isolation of OTA-producing fungal strains. **(A)** Tea samples from six different regions. **(B)** Chromatograms of OTA in dark tea sample. **(C)** *Aspergillus* fungal strains isolated from tea samples containing OTA. **(D)** Phylogenetic tree of *A. niger* AN3 based on combined sequences of *BenA*, *CaM*, and *RPB2* gene. **(E)** OTA-producing fungi, *A. niger* AN3 and Chromatograms of OTA-producing fungi.

Chromatograms of OTA in dark tea sample are shown in Figure 2B. The determination coefficient was *R*^2^ > 0.9991, and the regression equation (OTA concentration = 6.53 × 10^−2^ × peak area-0.0369) showed suitable linearity from 1 to 30 ng/ml. LOD and LOQ of OTA using the current method were 0.39 and 1.30 μg/kg, respectively. Under the different spiked concentrations, the recoveries by the method used in this study ranged from 78.8 ± 1.3 to 103.6 ± 9.4%. It is shown that the present method was suitable for fermented tea sample determination.

### OTA concentration in dark tea

A total of 228 dark tea samples from six regions (Figure 2A) were tested (Supplementary Table S1), and 21 samples contained OTA, as shown in Table 1; the occurrence of OTA was 9.2% (21/228), with concentrations ranging from 2.51 ± 0.16 to 12.62 ± 0.72 μg/kg.

**Table 1:** OTA concentrations above LOQ of dark tea samples from different regions and years.

No.	Year	Region	Type	Concentration (μg/kg)
GX38	2016	Guangxi	Liupao tea	4.59 ± 3.02
HN2	2019	Hunan	Dark tea	9.75 ± 1.65
HB10	2000	Hunan	Dark tea	2.63 ± 0.67
HB13	2015	Hunan	Fu brick tea	6.80 ± 0.59
HN16	2014	Hunan	Tianjian tea	3.92 ± 0.49
HN17	2017	Hunan	Qing brick tea	2.79 ± 0.61
HN22	2018	Hunan	Fu brick tea	4.72 ± 0.24
HN24	2012	Hunan	Fu brick tea	2.76 ± 0.36
SC9	2014	Sichuan	Kang brick tea	4.61 ± 0.70
SC12	2016	Sichuan	Jinjian tea	5.38 ± 2.47
SC13	2016	Sichuan	Kang brick tea	9.83 ± 1.54
SC15	2018	Sichuan	Kang brick tea	7.60 ± 1.00
SC18	2014	Sichuan	Jinjian tea	5.75 ± 2.01
SC24	2017	Sichuan	Kang brick tea	3.90 ± 0.12
SC30	2015	Sichuan	Kang brick tea	4.55 ± 0.62
SC36	2017	Sichuan	Dark tea	7.74 ± 0.94
SC38	2021	Sichuan	Dark tea	5.36 ± 0.29
SC39	2021	Sichuan	Kang brick tea	12.62 ± 0.72
SC44	2021	Sichuan	Tibetan tea	5.61 ± 2.88
SX1	2018	Shannxi	Fu brick tea	3.52 ± 0.07
YN30	2020	Yunnan	Ripe Pu-erh tea	2.51 ± 0.16

### Dark tea consumption

A total of 236 males and 210 females participated in the questionnaire survey. We grouped consumers by gender and age into two groups for males and two groups for females and five age groups: <21 years, 21–30 years, 31–40 years, 41–50 years, and 50 years and above. The average total black tea consumption was 7.02 g per day. Weight and dark tea consumption data for each group are presented in Supplementary Table S2.

### Risk assessment

#### Deterministic risk assessment

The OTA deterministic risk assessment was based on OTA exposure contamination data from 228 dark tea samples and tea consumption data from 446 respondents obtained in this study. In the deterministic risk assessment, consumers with average and heavy tea consumption (95% tea consumption) were considered to have average and heavy OTA exposure, respectively. In addition, the mean of all tea samples containing OTA levels in this study and the maximum OTA contamination level was considered extreme cases for exposure assessment. The WHO PMTDI value for OTA is 0.0143 μg/kg b.w. (JECFA, 2002). The risk factor was calculated by comparing the exposure data with the WHO-recommended PMTDI values. The deterministic potential neoplastic risk was assessed based on the exposure calculations, and the detailed results are shown in Table 2.

**Table 2:** Deterministic and probabilistic risk assessments of OTA in dark tea.

Group	Average-intake consumers					Heavy-intake consumers				
	LB	MB	UB	AVE	MAX	LB	MB	UB	AVE	MAX
**(A) Deterministic risk assessment of OTA in dark tea**										
All	4.005 × 10^−3^	5.387 × 10^−3^	6.769 × 10^−3^	4.348 × 10^−2^	9.864 × 10^−2^	1.142 × 10^−2^	1.536 × 10^−2^	1.930 × 10^−2^	1.239 × 10^−1^	2.812 × 10^−1^
Male	**4.230 × 10** ^−3^	**5.690 × 10** ^−3^	**7.151 × 10** ^−3^	**4.593 × 10** ^−2^	**1.042 × 10** ^−1^	**2.035 × 10** ^−3^	**2.738 × 10** ^−3^	**3.441 × 10** ^−3^	**2.210 × 10** ^−2^	**5.014 × 10** ^−2^
Female	3.675 × 10^−3^	4.944 × 10^−3^	6.212 × 10^−3^	3.990 × 10^−2^	9.053 × 10^−2^	1.322 × 10^−2^	1.779 × 10^−2^	2.235 × 10^−2^	1.436 × 10^−1^	3.257 × 10^−1^
<21	1.426 × 10^−3^	1.918 × 10^−3^	2.410 × 10^−3^	1.548 × 10^−2^	3.512 × 10^−2^	6.394 × 10^−3^	8.601 × 10^−3^	1.081 × 10^−2^	6.942 × 10^−2^	1.575 × 10^−1^
21–30	2.586 × 10^−3^	3.479 × 10^−3^	4.371 × 10^−3^	2.808 × 10^−2^	6.370 × 10^−2^	1.126 × 10^−2^	1.515 × 10^−2^	1.904 × 10^−2^	1.223 × 10^−1^	2.775 × 10^−1^
31–40	4.566 × 10^−3^	6.142 × 10^−3^	7.718 × 10^−3^	4.957 × 10^−2^	1.125 × 10^−1^	**1.130 × 10** ^−2^	**1.520 × 10** ^−2^	**1.910 × 10** ^−2^	**1.227 × 10** ^−1^	**2.783 × 10** ^−1^
41–50	5.044 × 10^−3^	6.786 × 10^−3^	8.527 × 10^−3^	5.477 × 10^−2^	1.243 × 10^−1^	1.051 × 10^−2^	1.414 × 10^−2^	1.777 × 10^−2^	1.141 × 10^−1^	2.589 × 10^−1^
>50	**5.313 × 10** ^−3^	**7.147 × 10** ^−3^	**8.980 × 10** ^−3^	**5.768 × 10** ^−2^	**1.309 × 10** ^−1^	1.108 × 10^−2^	1.490 × 10^−2^	1.873 × 10^−2^	1.203 × 10^−1^	2.729 × 10^−1^
**(B) Probabilistic risk assessment of OTA in dark tea**										
all	2.106 × 10^−2^	2.137 × 10^−2^	2.169 × 10^−2^	4.322 × 10^−2^	9.805 × 10^−2^	4.783 × 10^−2^	4.853 × 10^−2^	4.927 × 10^−2^	9.815 × 10^−2^	2.227 × 10^−1^
male	**2.113 × 10** ^−2^	**2.143 × 10** ^−2^	**2.176 × 10** ^−2^	**4.335 × 10** ^−2^	**9.836 × 10** ^−2^	4.524 × 10^−2^	4.589 × 10^−2^	4.659 × 10^−2^	9.282 × 10^−2^	2.106 × 10^−1^
female	2.043 × 10^−2^	2.072 × 10^−2^	2.104 × 10^−2^	4.191 × 10^−2^	9.509 × 10^−2^	**4.902 × 10** ^−2^	**4.973 × 10** ^−2^	**5.049 × 10** ^−2^	**1.006 × 10** ^−1^	**2.282 × 10** ^−1^
<21	8.623 × 10^−3^	8.748 × 10^−3^	8.882 × 10^−3^	1.769 × 10^−2^	4.014 × 10^−2^	2.864 × 10^−2^	2.906 × 10^−2^	2.950 × 10^−2^	5.877 × 10^−2^	1.333 × 10^−1^
21–30	7.017 × 10^−3^	7.119 × 10^−3^	7.227 × 10^−3^	1.440 × 10^−2^	3.267 × 10^−2^	2.001 × 10^−2^	2.030 × 10^−2^	2.061 × 10^−2^	4.106 × 10^−2^	9.315 × 10^−2^
31–40	2.070 × 10^−2^	2.101 × 10^−2^	2.133 × 10^−2^	4.249 × 10^−2^	9.639 × 10^−2^	4.944 × 10^−2^	5.016 × 10^−2^	5.092 × 10^−2^	1.014 × 10^−1^	2.302 × 10^−1^
41–50	2.406 × 10^−2^	2.441 × 10^−2^	2.478 × 10^−2^	4.937 × 10^−2^	1.120 × 10^−1^	4.821 × 10^−2^	4.892 × 10^−2^	4.966 × 10^−2^	9.893 × 10^−2^	2.245 × 10^−1^
>50	**2.522 × 10** ^−2^	**2.559 × 10** ^−2^	**2.598 × 10** ^−2^	**5.176 × 10** ^−2^	**1.174 × 10** ^−1^	**5.131 × 10** ^−2^	**5.205 × 10** ^−2^	**5.285 × 10** ^−2^	**1.053 × 10** ^−1^	**2.389 × 10** ^−1^

Deterministic risk assessment of OTA in dark tea. Average-intake consumers: average tea consumption, heavy-intake consumers: 95th percentile of tea consumption. LB (lower bound) indicates that 0 was used as the contamination value for undetected samples in the risk assessment. MB (middle bound) indicates that the 1/2 LOD value of the corresponding study was used as the contamination value for undetected samples in the risk assessment. UB (upper bound) indicates that the LOD value of the corresponding study was used as the contamination value for undetected samples in the risk assessment. AVE, the average contamination value in this study, was selected as the contamination value for the extreme-case risk assessment. Max, the maximum contamination value in this study, was selected as the contamination value for terrible case risk assessment. Values with bold font are the maximal HQ to each comparison group.

The results of the deterministic risk assessment indicated that the maximal HQ values for lower bound (LB), middle bound (MB), and upper bound (UB) were 5.313 × 10^−3^, 7.147 × 10^−3^, and 8.980 × 10^−3^ for average-intake consumers (>50 age group), respectively. The maximal HQ values for LB, MB, and UB were 1.130 × 10^−2^, 1.520 × 10^−2^, and 1.910 × 10^−2^, respectively, for heavy-intake consumers (31–40 years).

The maximal HQ values for all the groups were below 1.0, indicating no neoplastic risk. In the two extremes of exposure, the mean and maximal OTA levels in all tea samples containing OTA in this study, the maximal HQ values were 5.768 × 10^−2^ and 1.309 × 10^−1^, respectively, both below 1.0, indicating no neoplastic risk.

#### Probabilistic risk assessment

Probabilistic estimation was performed using the OTA pollution exposure concentrations, tea consumption, and consumer weight. First, the best-fit distributions of the exposure factors for different scenarios were examined. As shown in Supplementary Table S3, the best-fit distributions for the OTA contamination concentrations under the LB, MB, and UB scenarios were all minimum extreme value distributions, the best-fit distribution for body weight was a gamma distribution, and the best-fit distribution for daily tea consumption was a maximum extreme value distribution. Probabilistic risks were estimated for each group and the results are presented in Table 2.

The results of the neoplastic risk assessment showed that the HQ values for LB, MB, and UB were 5.131 × 10^−2^, 5.205 × 10^−2^, and 5.285 × 10^−2^, respectively, for heavy-intake consumers. The HQ values of 1.053 × 10^−1^ and 2.389 × 10^−1^ for heavy-intake consumers for the two extreme OTA exposure scenarios of average and maximum contamination concentrations, respectively, were also below 1.0, indicating no neoplastic risk. The results of deterministic and probabilistic risk assessments indicate that there was no OTA neoplastic risk from dark tea consumption. Sensitivity analysis showed that OTA contamination was the first key factor in the exposure assessment, with an *R* of 0.496 for the UB case and 0.472 for the UB case, with daily tea intake as the second influencing factor, as shown in Supplementary Table S4.

### Isolation of OTA-producing fungi

A total of 12 *Aspergillus* fungal strains (Figure 2C) were isolated from tea samples containing OTA from six different regions of China (Sichuan, Hunan, Yunnan, Hubei, Shaanxi, and Guangxi). Only one strain (AN3) has the capacity to produce OTA. The maximum likelihood tree based on combined sequences of *BenA*, *CaM*, and *RPB2* gene (Figure 2D) showed that AN3 was closely related to *Aspergillus niger* (the partial *BenA*, *CaM*, and *RPB2* gene sequences were deposited in GenBank under accession numbers OP682803, OP682804, and OP682805, respectively). Therefore, the AN3 was identified as *Aspergillus niger*. The Chromatograms of OTA-producing fungi are shown in Figure 2E.

### Inhibitory effects of TPs and EGCG on growth and toxicity production

**Figure 3:**
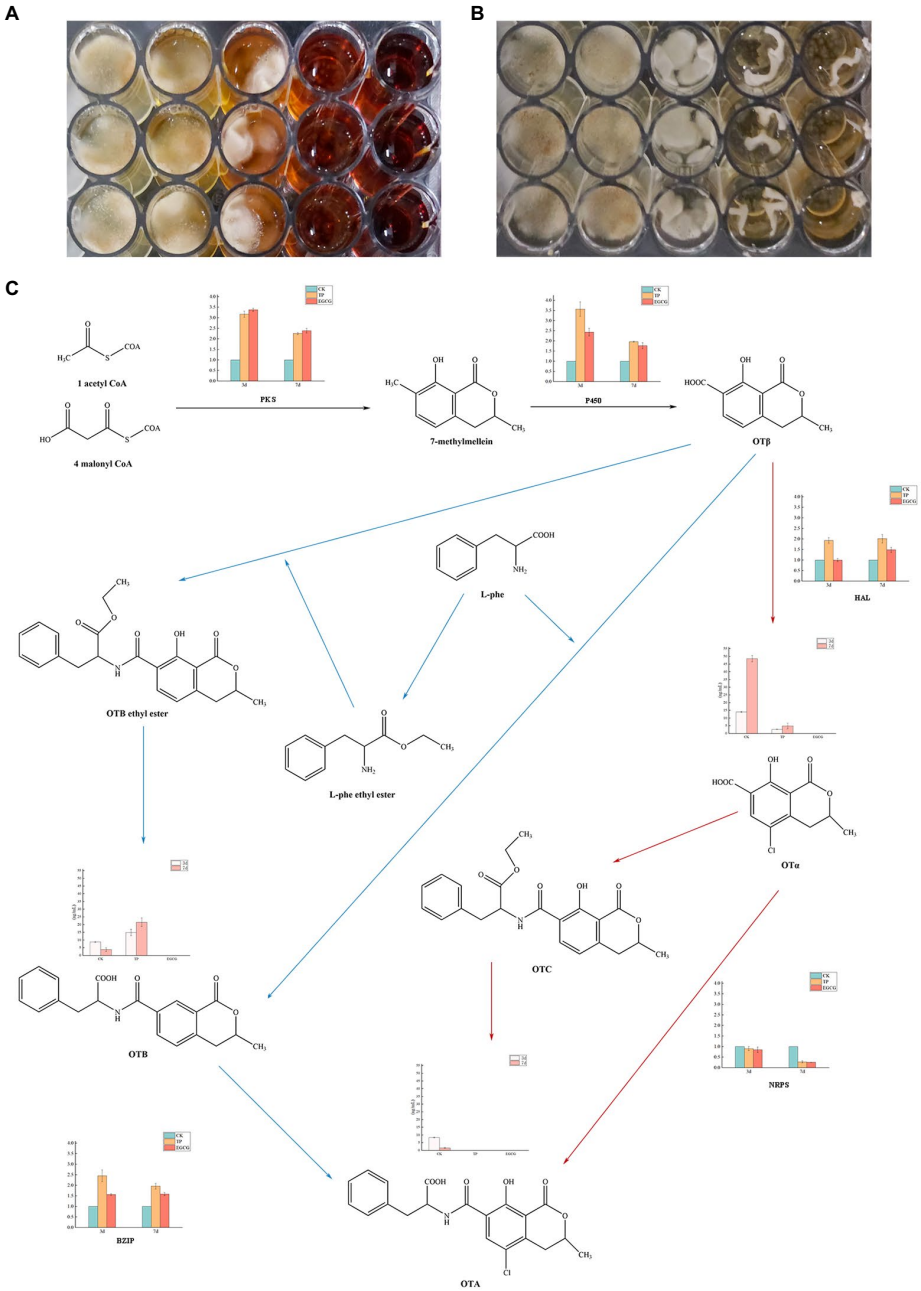
Inhibitory effects of TPs and EGCG on *Aspergillus niger*. **(A)** Growth of *A. niger* AN3 in medium containing TPs. **(B)** Growth of *A. niger* AN3 in medium containing EGCG. **(C)** OTA synthesis pathway and expression of OTA synthesis-related genes and concentrations of OTα, OTA, and OTB in *A. niger* AN3 (*BZIP*, a pathway-specific regulator that controls OTA production by regulating the *PKS*, *P450*, *HAL*, and *NRPS*).

The growth of *A. niger* AN3 after the addition of different concentrations of TPs and EGCG in the CYB medium for 7 days is shown in Figure 3. The inhibition of *A. niger* AN3 growth at low concentrations (0, 10, and 25 mg/ml) of TPs was not significant. The growth of *A. niger* AN3 was strongly inhibited at higher concentrations of TPs (50 mg/ml), and it was inhibited almost completely at higher concentrations of TPs (75 mg/ml). The growth of *A. niger* AN3 was not significantly inhibited at low concentrations of EGCG (0, 10, and 25 mg/ml), whereas it was inhibited at higher concentrations of EGCG (50 and 75 mg/ml) but not completely, and *A. niger* AN3 was still able to grow at higher concentrations of EGCG.

The growth of *A. niger* AN3 was inhibited with increasing concentrations of TPs and EGCG. In the detection of OTA production, we found that no OTA was detected in *A. niger* AN3 under the treatment culture conditions with TPs and EGCG, both at low and high concentrations. After 3 and 7 days of incubation with the addition of TPs and EGCG, although *A. niger* AN3 was able to grow at lower concentrations, OTA production was not detected.

### Mechanism of inhibition of toxicity production by TPs and EGCG

The concentrations of OTα, OTA, and OTB in the cultures after 3 and 7 days of incubation are presented in Table 3. We found no detectable production of OTA under either TPs or EGCG treatment. Under TPs treatment conditions, the OTα content at 7 days of culture was higher than that at 3 days of culture. However, we found that OTα levels were lower under TPs treatment than in the blank control, and no OTα production was detected under EGCG treatment. We speculated that this may be due to the inhibition of OTα production by TPs and EGCG through the inhibition of *A. niger* AN3 growth and its conversion to other substances, such as OTB and OTB ethyl ester.

**Table 3:** Concentrations of OTα, OTA, and OTB after treatment with different concentrations of TPs and EGCG.

Treatment	Concentrations (mg/ml)	OTα(ng/ml)	OTB(ng/ml)	OTA(ng/ml)
CK	/	48.54 ± 2.09	3.81 ± 1.21	1.55 ± 0.34
tea polyphenols	10	19.58 ± 1.40	13.44 ± 0.93	ND
	25	19.68 ± 1.32	2.94 ± 0.97	ND
	50	15.78 ± 0.67	ND	ND
	75	29.14 ± 2.16	ND	ND
EGCG	10	94.76 ± 2.57	4.75 ± 0.17	ND
	25	15.15 ± 7.89	2.60 ± 0.67	ND
	50	ND	ND	ND
	75	ND	ND	ND

CK represents a blank control without added tea polyphenols and EGCG.

Meanwhile, we observed that OTB levels at 7 days were higher than those at 3 days. However, unlike OTα, OTB levels were higher in the TPs treatment culture than in the blank control. This is probably due to the inhibition of OTα synthesis in favor of OTB. After EGCG treatment, OTα, OTA, and OTB were not detected. We speculated that EGCG may inhibit mycotoxin production by inhibiting their growth. We also found lower levels of OTA after 7 days incubation than after 3 days incubation, and Varga et al. (2000) suggested that *Aspergillus niger* may secrete enzymes to degrade OTA into other products, or in the case of nutrient deficiency, OTA is consumed as a supplementary nitrogen source.

The OTA synthesis pathway has not been completely resolved and only a few predicted pathways are available. The major OTA synthesis pathways postulated by Huff and Hamilton (1979) and Harris and Mantle (2001) are shown in Figure 3. We found a significant reduction (*p < 0.01*) in the relative expression of *NRPS* by real-time fluorescence quantification of genes critical for possible OTA synthesis in *A. niger* AN3. No OTA production was detected in any of the cultures, suggesting that TPs and EGCG inhibit the expression of *NRPS* and thus may suppress OTA toxin synthesis. Moreover, TPs and EGCG may inhibit the growth of *A. niger* AN3 through other pathways, thus suppressing OTA production.

## Discussion

Mycotoxin contamination in dark tea has been a topical issue for researchers and consumers in recent years, whether it is the presence of mycotoxins in dark tea affects people’s health or the residual mycotoxins having an impact on consumers’ health. Many studies have suggested the possibility of mycotoxin contamination in dark tea; aflatoxins (Wu J., 2013; Cui et al., 2020), ochratoxins (Liu et al., 2016; Mo et al., 2016), vomitoxins (Liu et al., 2017; Hu et al., 2019), and citrinins (Li et al., 2020) have been detected in dark tea samples. In contrast to other studies, 228 dark tea samples from different regions and years were investigated, demonstrating a wide and better representative origin, and 9.2% of the samples contained OTA. Haas et al. (2013) investigated 36 Ripe Pu-erh tea samples, and OTA was detected in four samples at concentrations of 0.65, 0.65, 14.8, and 94.7 μg/kg, with a detection rate of 11%. Mo et al. (2016) conducted a survey of 100 tea samples and four samples with OTA concentrations ranging from 0.22 to 0.44 μg/kg. Liu et al. (2016) surveyed 25 fermented tea samples, and one Hunan Fu brick tea sample was found to be contaminated by OTA at 4.2 μg/kg. Liu et al. analyzed 61 dark tea samples (Liu et al., 2017) using Quick Easy Cheap Effective Rugged Safe-ultra-high-performance liquid chromatography–tandem mass spectrometry and OTA was detected in five samples, including two samples of Ripe Pu-erh tea (6.7 and 2.5 μg/kg), one sample of Hunan dark tea (4.0 μg/kg), and two samples of Guangxi Liu Bao tea (0.9 and 1.3 μg/kg). Another more comprehensive survey involving 108 dark tea samples was conducted by Ye et al. (2020), which showed that five samples were contaminated by OTA with an average concentration of 0.66 μg/kg and a maximum concentration of 36.4 μg/kg. The presence of OTA in these dark tea samples could be attributed to two reasons. First, the dark tea may have been infested with exogenous ochratoxigenic fungi during the fermentation process. Second, some of the dark tea samples were stored for a long period or under unfavorable environmental conditions during the storage process, which contributed to the OTA contamination of dark tea.

There are some differences between the results of this study and those of others, which may be due to differences in the source of the samples or the detection methods. Combined with the above findings, it can be inferred that dark tea can be contaminated by OTA, which poses a potential health hazard to consumers. Therefore, it is expected that more researchers will conduct assessments on OTA contamination in dark tea.

Despite the risk of mycotoxin contamination in dark tea, studies by Cui et al. (2020), Ye et al. (2020), and Yan et al. (2021) show that the consumption of dark tea does not pose a significant health risk to consumers. Yan et al. (2021) assessed neoplastic risk based on the results of the Malir study (OTA level of 250 μg/kg; Malir et al., 2014), and only consumers with moderate and heavy tea consumption had maximal HQ values close to 1 or ≥1, indicating a potential neoplastic risk. However, the results of the study used a very extreme scenario. With the exception of Malir’s findings (Malir et al., 2014), other researchers have reported OTA exposure values well below 250 μg/kg. The results of this study are similar to those of other researchers, which also showed that OTA exposure from dark tea consumption posed no health risks to the population. However, there are limitations to the dark tea sample and consumer tea consumption data in this study. It is expected that more researchers will focus on mycotoxins in dark tea in the future to obtain better and more accurate results.

Some studies show that TPs have a significant inhibitory effect on aflatoxin synthesis (Mo et al., 2013; Wu Q., 2013), and the presence of antioxidants also inhibits the synthesis of aflatoxin B_1_ (Zhao, 2014). Zhang (2014) suggested that quercetin, a polyphenol in tea leaves, is an active factor in inhibiting the growth and toxicity of *Aspergillus flavus*, and that this substance induces downregulation of the expression of *AflS* and *AflR* by activating the antioxidant system transcription factor *Yap1*. Few studies have been conducted on the inhibition of OTA production by bioactive components. The results of Jiang et al. (2022) show that five genes in the OTA biosynthesis gene cluster, in particular the global regulator *LaeA* that regulates OTA biosynthesis, was markedly downregulated by eugenol. In this study, the evaluation of the inhibitory ability of TPs and EGCG at different concentrations in a co-cultivation system with *A. niger* AN3 showed that TPs and EGCG have significant inhibitory effects on *A. niger* AN3 under liquid culture conditions. *A. niger* AN3 was able to grow at lower concentrations but without OTA production, which prompted us to question whether TPs and EGCG inhibited the expression of key genes related to OTA synthesis in *A. niger* AN3. Further studies revealed that TPs and EGCG may have inhibited the production of OTA by *A. niger* AN3 by suppressing the expression of *NRPS*, a key gene for OTA synthesis. In this study, TPs and EGCG were found to inhibit *NRPS* expression in *A. niger* AN3; however, the principle of inhibition has not yet been identified, and further studies are required. Further research on the inhibition of OTA synthesis by biologically active ingredients is expected. Effective methods to control OTA contamination in tea include preventing toxin production and degrading pre-existing fungal toxins. Strictly regulated management during tea production and processing is the best way to prevent OTA contamination; however, if contamination has already occurred, appropriate methods need to be employed to remove it or reduce its toxicity. The results of this study indicate that TPs and EGCG can inhibit the growth and toxicity of *A. niger* AN3, providing new insights and ideas for the prevention of OTA contamination.

## Conclusion

In recent years, dark tea consumption has gradually increased. However, the contamination of dark tea with mycotoxins has received considerable attention from consumers and researchers. In this study, a rapid HPLC method using OTA was used to determine mycotoxins in dark tea with superior sensitivity and satisfactory recovery. A validated method was used to determine the contamination levels of OTA in 228 Chinese dark teas, and the proportion of tea samples containing OTA was 9.2%. The results of the deterministic and probabilistic risk assessments indicated that there was no neoplastic risk to human health from OTA exposure due to dark tea consumption. No consumption risk was observed for Chinese dark tea, and the results are important for consumers in countries that enjoy Chinese dark tea. Furthermore, 12 *Aspergillus* spp. strains were isolated from tea samples containing OTA, where only one strain of *A. niger* had the ability to produce OTA. The inhibitory effects of TPs and EGCG on growth and OTA production may provide new ideas for the detoxification of ochratoxins, but the most effective way to avoid OTA contamination of dark tea is to avoid the infestation of harmful microorganisms at the source during production, processing, and storage.

## Data availability statement

The data presented in the study are deposited in the https://www.ncbi.nlm.nih.gov/ repository, accession number: OP682803, OP682804, and OP682805.

## Author contributions

Y-qZ: conceptualization, methodology, formal analysis, and writing of the original draft. W-bJ: software investigation. S-yL and LX: software and validation. WC: methodology and validation. YZ and M-ZZ: writing—review and editing. WX: conceptualization, methodology, formal analysis, writing—review and editing, and funding acquisition. All authors contributed to the article and approved the submitted version.

## Funding

This work was supported by the National Key R&D Program of China (2019YFC0840503), Sichuan Province S&T Project (2021ZHFP0021), and Sichuan Agricultural University I&T Program (202210626034), Natural Science Foundation of China (32002095 and 32172217), Hunan “Three Top” Innovative Talents Project (2022RC1142), Natural Science Foundation of Hunan Province for Outstanding Young Scholars (2022JJ20028), and Changsha City Outstanding Innovative Youth Training Program (kq2107015).

## Conflict of interest

The authors declare that the research was conducted in the absence of any commercial or financial relationships that could be construed as a potential conflict of interest.

## Publisher’s note

All claims expressed in this article are solely those of the authors and do not necessarily represent those of their affiliated organizations, or those of the publisher, the editors and the reviewers. Any product that may be evaluated in this article, or claim that may be made by its manufacturer, is not guaranteed or endorsed by the publisher.

## Supplementary material

The Supplementary material for this article can be found online at: https://www.frontiersin.org/articles/10.3389/fmicb.2022.1073950/full#supplementary-material
