# Supplementary material for: Dietary assessment of ochratoxin A in Chinese dark tea and inhibitory effects of tea polyphenols on ochratoxigenic Aspergillus niger
Source: Front Microbiol. 2022 Dec 6;13:1073950. doi: 10.3389/fmicb.2022.1073950 (PMC9763595; doi:10.3389/fmicb.2022.1073950)
Supplement: Supplementary file 1 [file Data_Sheet_1.docx]

Table S1. Information on the tea samples and OTA concentrations.

| No. | year | region | type | OTA concentration(μg/kg) | No. | year | region | type | OTA concentration(μg/kg) |
| --- | --- | --- | --- | --- | --- | --- | --- | --- | --- |
| SC1 | 2011 | Sichuan | Kang brick tea | ND | SC15 | 2018 | Sichuan | Kang brick tea | 7.60±1.00 |
| SC2 | 2017 | Sichuan | Jinjian tea | ND | SC16 | 2017 | Sichuan | Kang brick tea | ND |
| SC3 | 2020 | Sichuan | Fu brick tea | ND | SC17 | 2018 | Sichuan | Jinjian tea | ND |
| SC4 | 2020 | Sichuan | Fu brick tea | ND | SC18 | 2014 | Sichuan | Jinjian tea | 5.75±2.01 |
| SC5 | 2020 | Sichuan | Fu brick tea | ND | SC19 | 2018 | Sichuan | Jinjian tea | ND |
| SC6 | 2017 | Sichuan | Kang brick tea | ND | SC20 | 2020 | Sichuan | Dark tea | ND |
| SC7 | 2016 | Sichuan | Kang brick tea | ND | SC21 | 2016 | Sichuan | Kang brick tea | ND |
| SC8 | 2017 | Sichuan | Kang brick tea | ND | SC22 | 2017 | Sichuan | Kang brick tea | ND |
| SC9 | 2014 | Sichuan | Kang brick tea | 4.61±0.70 | SC23 | 2021 | Sichuan | Kang brick tea | ND |
| SC10 | 2015 | Sichuan | Dark tea | ND | SC24 | 2017 | Sichuan | Kang brick tea | 3.90±0.12 |
| SC11 | 2017 | Sichuan | Tibetan tea | ND | SC25 | 2017 | Sichuan | Kang brick tea | ND |
| SC12 | 2016 | Sichuan | Jinjian tea | 5.38±2.47 | SC26 | 2016 | Sichuan | Jinjian tea | ND |
| SC13 | 2016 | Sichuan | Kang brick tea | 9.83±1.54 | SC27 | 2019 | Sichuan | Kang brick tea | ND |
| SC14 | 2017 | Sichuan | Qing brick tea | ND | SC28 | 2015 | Sichuan | Jinjian tea | ND |

| **Table S1 (continued)** Information on tea samples and its OTA concentration | | | | | | | | | |  |
| --- | --- | --- | --- | --- | --- | --- | --- | --- | --- | --- |
| No. | year | region | type | OTA concentration(μg/kg) | No. | year | region | type | OTA concentration(μg/kg) | |
| SC29 | 2016 | Sichuan | Jinjian tea | ND | SC43 | 2021 | Sichuan | Fu brick tea | ND |  |
| SC30 | 2015 | Sichuan | Kang brick tea | 4.55±0.62 | SC44 | 2021 | Sichuan | Tibetan tea | 5.61±2.88 |  |
| SC31 | 2017 | Sichuan | Kang brick tea | ND | SC45 | 2021 | Sichuan | Tibetan tea | ND |  |
| SC32 | 2018 | Sichuan | Jinjian tea | ND | SC46 | 2021 | Sichuan | Tibetan tea | ND |  |
| SC33 | 2015 | Sichuan | Jinjian tea | ND | SC47 | 2021 | Sichuan | Tibetan tea | ND |  |
| SC34 | 2012 | Sichuan | Tibetan tea | ND | HN1 | 2012 | Hunan | Dark tea | ND |  |
| SC35 | 2017 | Sichuan | Kang brick tea | ND | HN2 | 2019 | Hunan | Dark tea | 9.75±1.65 |  |
| SC36 | 2017 | Sichuan | Dark tea | 7.74±0.94 | HN3 | 1981 | Hunan | Dark tea | ND |  |
| SC37 | 2018 | Sichuan | Kang brick tea | ND | HN4 | 2015 | Hunan | Fu brick tea | ND |  |
| SC38 | 2021 | Sichuan | Dark tea | 5.36±0.29 | HN5 | 2018 | Hunan | Fu brick tea | ND |  |
| SC39 | 2021 | Sichuan | Kang brick tea | 12.62±0.72 | HN6 | 2021 | Hunan | Fu brick tea | ND |  |
| SC40 | 2021 | Sichuan | Jinjian tea | ND | HN7 | 2018 | Hunan | Dark tea | ND |  |
| SC41 | 2021 | Sichuan | Kang brick tea | ND | HN8 | 2013 | Hunan | Fu brick tea | ND |  |
| SC42 | 2020 | Sichuan | Kang brick tea | ND | HN9 | 2015 | Hunan | Fu brick tea | ND |  |
| HN10 | 2017 | Hunan | Qianliang tea | ND | HN24 | 2012 | Hunan | Fu brick tea | 2.76±0.36 |  |
| HN11 | 2015 | Hunan | Hei brick tea | ND | HN25 | 2018 | Hunan | Fu brick tea | ND |  |
| HN12 | 2018 | Hunan | Qianliang tea | ND | HN26 | 2012 | Hunan | Fu brick tea | ND |  |
| HN13 | 2016 | Hunan | Fu brick tea | ND | HN27 | 2011 | Hunan | Hei brick tea | ND |  |
| HN14 | 2019 | Hunan | Qianliang tea | ND | HN28 | 2016 | Hunan | Qianliang tea | ND |  |
| HN15 | 2014 | Hunan | Fu brick tea | ND | HN29 | 2019 | Hunan | Qianliang tea | ND |  |
| HN16 | 2014 | Hunan | Dark tea | 3.92±0.49 | HN30 | 2016 | Hunan | Fu brick tea | ND |  |
| HN17 | 2017 | Hunan | Qing brick tea | 2.78±0.61 | HN31 | 2018 | Hunan | Hei brick tea | ND |  |
| HN18 | 2019 | Hunan | Dark tea | ND | HN32 | 2016 | Hunan | Qianliang tea | ND |  |
| HN19 | 2015 | Hunan | Fu brick tea | ND | HN33 | 2021 | Hunan | Hei brick tea | ND |  |
| HN20 | 2012 | Hunan | Fu brick tea | ND | HN34 | 2016 | Hunan | Fu brick tea | ND |  |
| HN21 | 2020 | Hunan | Qianliang tea | ND | HN35 | 2018 | Hunan | Fu brick tea | ND |  |
| HN22 | 2018 | Hunan | Fu brick tea | 4.72±0.24 | HN36 | 2018 | Hunan | Qianliang tea | ND |  |
| HN23 | 2014 | Hunan | Qianliang tea | ND | HN37 | 2007 | Hunan | Fu brick tea | ND |  |
| HN38 | 2021 | Hunan | Fu brick tea | ND | YN2 | 2015 | Yunnan | Ripe Pu-erh tea | ND |  |
| HN39 | 2011 | Hunan | Fu brick tea | ND | YN3 | 2012 | Yunnan | Ripe Pu-erh tea | ND |  |
| HN40 | 2016 | Hunan | Fu brick tea | ND | YN4 | 2015 | Yunnan | Ripe Pu-erh tea | ND |  |
| HN41 | 2017 | Hunan | Dark tea | ND | YN5 | 2004 | Yunnan | Ripe Pu-erh tea | ND |  |
| HN42 | 2012 | Hunan | Qianliang tea | ND | YN6 | 2018 | Yunnan | Ripe Pu-erh tea | ND |  |
| HN43 | 2011 | Hunan | Dark tea | ND | YN7 | 2003 | Yunnan | Ripe Pu-erh tea | ND |  |
| HN44 | 2016 | Hunan | Fu brick tea | ND | YN8 | 2012 | Yunnan | Ripe Pu-erh tea | ND |  |
| HN45 | 2018 | Hunan | Qianliang tea | ND | YN9 | 2016 | Yunnan | Ripe Pu-erh tea | ND |  |
| HN46 | 2016 | Hunan | Fu brick tea | ND | YN10 | 2016 | Yunnan | Ripe Pu-erh tea | ND |  |
| HN47 | 2015 | Hunan | Fu brick tea | ND | YN11 | 2018 | Yunnan | Ripe Pu-erh tea | ND |  |
| HN48 | 2015 | Hunan | Fu brick tea | ND | YN12 | 2018 | Yunnan | Ripe Pu-erh tea | ND |  |
| HN49 | 2010 | Hunan | Fu brick tea | ND | YN13 | 2016 | Yunnan | Ripe Pu-erh tea | ND |  |
| HN50 | 2015 | Hunan | Qianliang tea | ND | YN14 | 2017 | Yunnan | Ripe Pu-erh tea | ND |  |
| YN1 | 2019 | Yunnan | Ripe Pu-erh tea | ND | YN15 | 2010 | Yunnan | Ripe Pu-erh tea | ND |  |
| YN16 | 2015 | Yunnan | Ripe Pu-erh tea | ND | YN30 | 2020 | Yunnan | Ripe Pu-erh tea | 2.51±0.16 |  |
| YN17 | 2008 | Yunnan | Ripe Pu-erh tea | ND | YN31 | 1998 | Yunnan | Ripe Pu-erh tea | ND |  |
| YN18 | 2008 | Yunnan | Ripe Pu-erh tea | ND | YN32 | 2018 | Yunnan | Ripe Pu-erh tea | ND |  |
| YN19 | 2008 | Yunnan | Ripe Pu-erh tea | ND | YN33 | 2009 | Yunnan | Ripe Pu-erh tea | ND |  |
| YN20 | 2014 | Yunnan | Ripe Pu-erh tea | ND | YN34 | 2020 | Yunnan | Ripe Pu-erh tea | ND |  |
| YN21 | 2020 | Yunnan | Ripe Pu-erh tea | ND | YN35 | 2008 | Yunnan | Ripe Pu-erh tea | ND |  |
| YN22 | 2018 | Yunnan | Ripe Pu-erh tea | ND | YN36 | 2007 | Yunnan | Ripe Pu-erh tea | ND |  |
| YN23 | 1998 | Yunnan | Ripe Pu-erh tea | ND | YN37 | 2003 | Yunnan | Ripe Pu-erh tea | ND |  |
| YN24 | 2008 | Yunnan | Ripe Pu-erh tea | ND | HB1 | 2006 | Hubei | Qing brick tea | ND |  |
| YN25 | 2020 | Yunnan | Ripe Pu-erh tea | ND | HB2 | 2019 | Hubei | Dark tea | ND |  |
| YN26 | 2007 | Yunnan | Ripe Pu-erh tea | ND | HB3 | 2021 | Hubei | Qing brick tea | ND |  |
| YN27 | 2020 | Yunnan | Ripe Pu-erh tea | ND | HB4 | 2016 | Hubei | Dark tea | ND |  |
| YN28 | 1980 | Yunnan | Ripe Pu-erh tea | ND | HB5 | 2017 | Hubei | Qing brick tea | ND |  |
| YN29 | 2017 | Yunnan | Ripe Pu-erh tea | ND | HB6 | 2018 | Hubei | Dark tea | ND |  |
| HB7 | 2020 | Hubei | Qing brick tea | ND | HB21 | 2021 | Hubei | Dark tea | ND |  |
| HB8 | 2021 | Hubei | Dark tea | ND | SX1 | 2018 | Shaanxi | Fu brick tea | 3.52±0.07 |  |
| HB9 | 2018 | Hubei | Gong brick tea | ND | SX2 | 2018 | Shaanxi | Fu brick tea | ND |  |
| HB10 | 2000 | Hubei | Dark tea | 2.63±0.67 | SX3 | 2019 | Shaanxi | Fu brick tea | ND |  |
| HB11 | 2014 | Hubei | Qing brick tea | ND | SX4 | 2016 | Shaanxi | Fu brick tea | ND |  |
| HB12 | 2020 | Hubei | Qing brick tea | ND | SX5 | 2013 | Shaanxi | Fu brick tea | ND |  |
| HB13 | 2015 | Hubei | Qing brick tea | 6.80±0.59 | SX6 | 2015 | Shaanxi | Fu brick tea | ND |  |
| HB14 | 2016 | Hubei | Qing brick tea | ND | SX7 | 2018 | Shaanxi | Fu brick tea | ND |  |
| HB15 | 2018 | Hubei | Qing brick tea | ND | SX8 | 2016 | Shaanxi | Fu brick tea | ND |  |
| HB16 | 2009 | Hubei | Qing brick tea | ND | SX9 | 2016 | Shaanxi | Fu brick tea | ND |  |
| HB17 | 2020 | Hubei | Qing brick tea | ND | SX10 | 2016 | Shaanxi | Fu brick tea | ND |  |
| HB18 | 2010 | Hubei | Qing brick tea | ND | SX11 | 2017 | Shaanxi | Fu brick tea | ND |  |
| HB19 | 2020 | Hubei | Qing brick tea | ND | SX12 | 2015 | Shaanxi | Fu brick tea | ND |  |
| HB20 | 2017 | Hubei | Qing brick tea | ND | SX13 | 2019 | Shaanxi | Fu brick tea | ND |  |
| SX14 | 2019 | Shaanxi | Fu brick tea | ND | SX28 | 2017 | Shaanxi | Fu brick tea | ND |  |
| SX15 | 2017 | Shaanxi | Fu brick tea | ND | SX29 | 2018 | Shaanxi | Fu brick tea | ND |  |
| SX16 | 2020 | Shaanxi | Fu brick tea | ND | SX30 | 2017 | Shaanxi | Fu brick tea | ND |  |
| SX17 | 2015 | Shaanxi | Fu brick tea | ND | SX31 | 2018 | Shaanxi | Fu brick tea | ND |  |
| SX18 | 2019 | Shaanxi | Fu brick tea | ND | GX1 | 2013 | Guangxi | Liupao tea | ND |  |
| SX19 | 2018 | Shaanxi | Fu brick tea | ND | GX2 | 2011 | Guangxi | Liupao tea | ND |  |
| SX20 | 2016 | Shaanxi | Fu brick tea | ND | GX3 | 2018 | Guangxi | Liupao tea | ND |  |
| SX21 | 2016 | Shaanxi | Fu brick tea | ND | GX4 | 2017 | Guangxi | Liupao tea | ND |  |
| SX22 | 2018 | Shaanxi | Fu brick tea | ND | GX5 | 2019 | Guangxi | Liupao tea | ND |  |
| SX23 | 2018 | Shaanxi | Fu brick tea | ND | GX6 | 2010 | Guangxi | Liupao tea | ND |  |
| SX24 | 2014 | Shaanxi | Fu brick tea | ND | GX7 | 2014 | Guangxi | Liupao tea | ND |  |
| SX25 | 2018 | Shaanxi | Fu brick tea | ND | GX8 | 2014 | Guangxi | Liupao tea | ND |  |
| SX26 | 2019 | Shaanxi | Fu brick tea | ND | GX9 | 2006 | Guangxi | Liupao tea | ND |  |
| SX27 | 2016 | Shaanxi | Fu brick tea | ND | GX10 | 2009 | Guangxi | Liupao tea | ND |  |
| GX11 | 2017 | Guangxi | Liupao tea | ND | GX25 | 2017 | Guangxi | Liupao tea | ND |  |
| GX12 | 2015 | Guangxi | Liupao tea | ND | GX26 | 2018 | Guangxi | Liupao tea | ND |  |
| GX13 | 2016 | Guangxi | Liupao tea | ND | GX27 | 2018 | Guangxi | Liupao tea | ND |  |
| GX14 | 2016 | Guangxi | Liupao tea | ND | GX28 | 2011 | Guangxi | Liupao tea | ND |  |
| GX15 | 2019 | Guangxi | Liupao tea | ND | GX29 | 2018 | Guangxi | Liupao tea | ND |  |
| GX16 | 2017 | Guangxi | Liupao tea | ND | GX30 | 2017 | Guangxi | Liupao tea | ND |  |
| GX17 | 2016 | Guangxi | Liupao tea | ND | GX31 | 2014 | Guangxi | Liupao tea | ND |  |
| GX18 | 2013 | Guangxi | Liupao tea | ND | GX32 | 2016 | Guangxi | Liupao tea | ND |  |
| GX19 | 2018 | Guangxi | Liupao tea | ND | GX33 | 2015 | Guangxi | Liupao tea | ND |  |
| GX20 | 2010 | Guangxi | Liupao tea | ND | GX34 | 2021 | Guangxi | Liupao tea | ND |  |
| GX21 | 2011 | Guangxi | Liupao tea | ND | GX35 | 2016 | Guangxi | Liupao tea | ND |  |
| GX22 | 2018 | Guangxi | Liupao tea | ND | GX36 | 2004 | Guangxi | Liupao tea | ND |  |
| GX23 | 2014 | Guangxi | Liupao tea | ND | GX37 | 2013 | Guangxi | Liupao tea | ND |  |
| GX24 | 2015 | Guangxi | Liupao tea | ND | GX38 | 2016 | Guangxi | Liupao tea | 4.59±3.02 |  |
| GX39 | 2017 | Guangxi | Liupao tea | ND | GX41 | 2020 | Guangxi | Liupao tea | ND |  |
| GX40 | 2018 | Guangxi | Liupao tea | ND | GX42 | 2013 | Guangxi | Liupao tea | ND |  |

ND represents OTA concentrations of samples below the LOD.

**Table S2. Population body weight and dark tea consumption.**

| group | individuals (n) | body weight（kg） | daily tea intake（g） |
| --- | --- | --- | --- |
| <21 | 20 | 55.25 | 2.20 |
| 21-30 | 160 | 59.86 | 4.32 |
| 31-40 | 104 | 63.48 | 8.08 |
| 41-50 | 72 | 68.24 | 9.60 |
| >50 | 90 | 64.75 | 9.59 |
| male | 236 | 70.48 | 8.31 |
| female | 210 | 54.25 | 5.56 |
| all | 446 | 62.84 | 7.02 |

**Table S3. Best-fitting distributions of the major exposure factors.**

| Exposure factor | Scenario | Units | Distribution | Parameter values |
| --- | --- | --- | --- | --- |
| OTA contamination | LB | μg·kg^-1^ | minimum extreme | most probable value:1.65, scale:3.29 |
|  | UB | μg·kg^-1^ | minimum extreme | most probable value:1.80, scale:3.23 |
|  | UB | μg·kg^-1^ | minimum extreme | most probable value:1.95, scale:3.16 |
| body weight | - | kg | gamma | location:34.19, scale:5.69, shape:5.04 |
| daily tea intake | - | g·day^-1^ | maximum extreme | most probable value:4.20, scale:4.57 |

**Table S4. Sensitivity analysis on variable factors in Monte Carlo simulation.**

| group | OTA contamination | daily tea intake | body weight |
| --- | --- | --- | --- |
| LB | 50.9% | 46.0% | -3.1% |
| MB | 49.4% | 47.4% | -3.1% |
| UB | 49.6% | 47.2% | -3.2% |

LB (lower bound) indicates that 0 was used as the contamination value for undetected samples in the risk assessment. MB (middle bound) indicates that the 1/2 LOD value of the corresponding study was used as the contamination value for undetected samples in the risk assessment. UB (upper bound) indicates that the LOD value of the corresponding study was used as the contamination value for undetected samples in the risk assessment.
